# Validating the generic quality of life tool “QOL10” in a substance use disorder treatment cohort exposes a unique social construct

**DOI:** 10.1186/s12874-016-0163-x

**Published:** 2016-05-23

**Authors:** Ashley E. Muller, Svetlana Skurtveit, Thomas Clausen

**Affiliations:** Norwegian Centre for Addiction Research, Institute of Clinical Medicine, University of Oslo, Postbox 1171, Blindern, 0318 Oslo, Norway; Department of Pharmacoepidemiology, The Norwegian Institute of Public Health, Oslo, Norway; Addiction Unit, Sørlandet Hospital HF, Kristiansand, Norway

## Abstract

**Background:**

Generic quality of life (QoL) instruments provide important measures of self-reported wellbeing that can be compared across healthy and clinical populations. The aim of this analysis is to validate the ten-item QoL instrument “QOL10”, as well as to confirm the validity of the embedded “QOL5” questionnaire and single-item “QOL1” in measuring overall QoL among adults in a substance use disorder treatment study.

**Methods:**

We used exploratory factor analysis and measured internal and convergent validity of the QOL10 against the gold standard measure of the WHOQOL-BREF, in a subsample of 107 participants in a substance use disorder treatment study.

**Results:**

The QOL10 displayed internal and convergent validity to the gold standard measure. Factor analysis revealed a two-factor structure that can be interpreted as “social QoL”, containing items about relationships and social functioning, and “global QoL”, comprised of items about health, working ability, self-evaluation, and an overall QoL estimation.

**Conclusions:**

The QOL10 provides clinically useful and valid measures of social-related QoL and global QoL via two subscales. Interestingly, the QOL10’s social QoL measure, from the current sample, had little relationship to the analyzed groups previously reported to have differential global QoL: social QoL appears to be not only conceptually distinct from global QoL, but also to be less influenced by typical substance- and treatment-specific factors.

## Background

Quality of life is one of the most common patient-reported outcomes, as it utilizes patients’ perceptions and expectations to capture the lived experiences of disease and treatment [1]. In marginalized patient groups such as persons with a substance use disorder (SUD), using subjective information from patients instead of clinicians to plan and evaluate treatment modalities is not only empowering, but also an effective way of discovering the effects of disease status and treatment on a wide range of life areas [2]. Subjectivity is a prerequisite to a good QoL instrument, as subjective responses allow participants to evaluate “*their* life satisfaction, taking into account the balance between positive and negative as it was relevant to *their* individual experience rather than the researcher deciding” (p.8) [2].

Multidimensional QoL measures such as the World Health Organization’s WHOQOL-BREF include mental health, physical mental, social, and environmental dimensions [1, 3]. Research with the SUD population, however, often focuses exclusively on health-related QoL [4, 5]. This is despite qualitative findings that patients conceptualize QoL to be more about social inclusion and support than health [2, 6] and that SUD patients report worse social functioning than other chronic disease groups [7].

Validating a QoL instrument among the SUD population exposes the problem of a paucity of known groups, that is, few subgroups which consistently report differential QoL and can therefore be used to test the sensitivity of the instrument. While persons with a SUD report markedly lower QoL than those without, including those with other chronic diseases [8], few QoL-specific indicators among the SUD population have been established. Reviews have shown psychological distress to be the most consistent predictor of poor QoL, while substance use severity, primary substance type, age, and gender have unclear relationships to QoL [5, 9]. While less systematically published, QoL has been reported to be higher among treatment completers versus drop-outs or those only beginning treatment [10, 11], the physically active compared to inactive [12], and those who report social activity versus isolation [13].

Generic as opposed to disease-specific QoL instruments are particularly important because they utilize a wellness paradigm rather than a pathology paradigm, and correspond to a treatment focus of improved overall functioning and satisfaction with functioning rather than simply symptom reduction [14]. Because they are not limited to disease-related functioning, the estimations of QoL they produce can be compared across healthy and clinical populations and interventions.

The validation of an additional QoL instrument provides researchers with a different mechanism – and more opportunities – to explore the differences in QoL and ascertain the influence of new factors. The QOL10 is of interest because it can be implemented with low administrative burden, due to its short length and simple scoring instructions. We wished to validate the QOL10 among the SUD population in Norway with an analytic strategy that has been previously used to validate patient-reported outcome measures [15, 16]. Secondly we wanted to confirm the validity of the embedded QOL5 questionnaire and single-item QOL1.

## Methods

### Participants

Data for this validation study were contributed by a sub-sample of participants in the Norwegian Cohort of Patients in Opioid Maintenance Treatment (NorComt), recently described in Skjærvø et al. [17]. The NorComt study is a naturalistic, longitudinal study of persons in opioid maintenance treatment (OMT) aimed at exploring factors that impact treatment retention and quality of life. 549 individuals beginning either OMT or medication-free inpatient treatment in 21 participating facilities were recruited from 2012 to 2015. The only inclusion criterion into this study was recent admittance into a substance use disorder treatment facility, regardless of primary substance type(s).

Data in this paper came from 107 consecutive follow-up interviews with participants who had begun treatment, and entered the study, one year earlier. Participants did not have to be in any formal treatment at the time of the follow-up interviews. Participants’ sociodemographic, substance-related, and health variables are presented in Table 1, and data collection is further described in *Procedure*.

**Table 1:** Sample descriptives (*N* = 107)

	n or mean	% or SD
Demographics		
Age	34.3	9.6
Women	36	33.6 %
Single	78	77.2 %
Primary education or less	61	57.0 %
Unemployed	76	71.0 %
Substance-related variables		
Most used substance/medication, past 6 months.		
*None*	20	18.7 %
*Methadone/buprenorphine*	32	29.9 %
*Cannabis*	14	13.1 %
*Alcohol*	13	12.1 %
*Amphetamines*	9	8.4 %
*Heroin/opium*	5	4.7 %
*Benzodiazepines*	5	4.6 %
*Ecstasy, LSD, stimulants, cocaine, inhalants, or other addictive substances*	0	0 %
*Other not listed*	9	8.6 %
Polysubstance user	49	49.0 %
Injected within past four weeks	25	25.5 %
Current SUD treatment		
*None*	20	19.9 %
*Outpatient with OMT*	46	43.8 %
*Outpatient without OMT*	15	14.0 %
*Inpatient with OMT*	14	13.1 %
Any treatment attrition, past year	35	35.0 %
Health variables		
Additional chronic disease	71	66.4 %
Clinical anxiety symptoms	61	57.0 %
Clinical depression symptoms	60	56.1 %
Received psychiatric services, past year	55	50.9 %
Physically inactive	38	35.5 %

Demographic, substance-related, and health variables of the sample

*SUD* substance use disorder

*OMT* Opioid maintenance treatment

### Measures

#### Quality of life measures

The QOL10 is a ten-item general QoL instrument developed using Ventegodt et al’s clinical experience with the QOL5, an earlier, shorter tool they developed [18, 19]. The QOL10 has not yet been validated, but was built from the validated five-question QOL5 (questions 1–5) and the validated single-item measure of QoL called the QOL1 (question 10). An example item is question 9, “how is your working ability at the moment?” Responses were given on a 1–5 Likert scale of “very good”, “good”, “neither good nor poor”, “poor”, and “very poor” (items are listed in Table 2).

**Table 2:** Descriptive statistics of the “QOL10”, “QOL5”, and “QOL1”

		Responses [n (%)]							
	N	Very poor	Poor	Neither good nor poor	Good	Very good	Mean (s.e.m.)	Skewness	Kurtosis
*Items*									
1. How do you consider your physical health at the moment? (QOL5)	107	7 (6.5)	15 (14.0)	29 (27.1)	43 (39.3)	14 (13.1)	0.58 (.02)	-.54	-.25
2. How do you consider your mental health at the moment? (QOL5)	107	7 (6.5)	14 (13.1)	32 (29.9)	41 (37.4)	14 (13.1)	0.58 (.02)	-.57	-.15
3. How do you feel about yourself at the moment? (QOL5)	105	4 (3.7)	11 (10.3)	38 (36.2)	42 (40.0)	10 (9.3)	0.58 (.02)	-.54	.20
4. How are your relationships with your friends at the moment? (QOL5)	102	4 (3.9)	12 (11.8)	17 (15.9)	57 (55.9)	12 (11.8)	0.63 (.02)	−1.15	1.27
5. How is your relationship with your partner at the moment? (QOL5)	43	1 (2.3)	4 (9.3)	5 (11.6)	14 (32.6)	19 (44.2)	0.66 (.02)	-.48	-.13
6. How do you consider your ability to love at the moment?	107	4 (3.7)	6 (5.6)	13 (12.1)	38 (35.5)	46 (43.0)	0.72 (.02)	−1.25	.1.13
7. How do you consider your sexual functioning at the moment?	102	11 (10.8)	11 (10.8)	18 (17.6)	34 (33.3)	28 (26.2)	0.61 (.03)	-.68	-.59
8. How do you consider your social functioning at the moment?	106	5 (4.7)	13 (12.1)	31 (29.0)	35 (32.7)	22 (20.6)	0.61 (.02)	-.44	-.42
9. How is your working ability at the moment?	104	13 (12.5)	15 (14.0)	23 (21.5)	34 (32.7)	19 (18.3)	0.56 (.02)	-.40	-.85
10. How would you assess your quality of your life now? (QOL1)	107	5 (4.7)	25 (23.4)	36 (33.6)	31 (29.0)	10 (9.3)	0.53 (.02)	-.05	-.62
*Scores*									
QOL10	103						0.59 (0.01)		
QOL5	104						0.60 (0.01)		
QOL1	107						0.53 (0.02)		

Descriptive statistics (item responses and scale statistics) of the three quality of life tools, “QOL10”, “QOL5”, and “QOL1”

The gold standard measurement selected was the WHOQOL-BREF, a widely used and validated 26-item self-report tool suggested to be the most appropriate multidimensional tool for use among the SUD population [14, 20]. The WHOQOL-BREF produces four domain scores – physical health QoL via seven items, psychological health QoL via six items, social relationships QoL via three items, and environment QoL via eight items – as well as one single-item measure of overall QoL mirroring the QOL1 and one of general health. The WHOQOL-BREF uses a nearly identical Likert scale as the QOL10 but with higher numbers indicating positive responses. We present the four domain scores as well as the single item measuring overall QoL; the second single-item question measuring self-reported health was not considered relevant to this analysis and is not presented.

#### Demographic, health, and substance-related measures

Substance use and severity indicators were measured using excerpts from the EuropASI [21], a validated version of the Addiction Severity Index adapted for European use. The Hopkins Symptoms Checklist-25 measured symptom levels of clinical anxiety and depression, using a cut-off of 1.0 on the anxiety and depression scales [22]. Missing items in the HSCL-25 were replaced by the appropriate subscale mean. Dichotomized health variables defining groups previously reported to have differential QoL were self-reports of additional chronic somatic disease status (with or without a chronic disease, e.g., coronary heart disease, diabetes, or hepatitis C), mental health status (with or without symptoms of clinical anxiety, and with or without symptoms of clinical depression), physical inactivity (those who were or were not inactive), and psychiatric services utilization (those who received any psychiatric services in the past year versus those who did not) [23–26]. Sociodemographic variables included civil status (“single” or “married/partnered”), unemployment, and educational attainment of primary school or less.

### Procedure

The NorComt study collected data via structured, face-to-face interviews conducted by trained interviewers, once when participants entered treatment (baseline) and again one year later (follow-up). Interviews typically lasted 1.5 h. The QOL10 and embedded QOL5 and QOL1 were collected at baseline and follow-up. In order to provide us with QoL data from a validated tool that we could use to determine how best to analyze the similar, longitudinal data from the QOL10 in future analyses, the gold standard WHOQOL-BREF was administered to a sub-sample of 107 consecutive follow-up interviews.

Participants provided written informed consent to the NorComt study, and the Norwegian Regional Ethics Committee approved the project and this subanalysis.

### Statistical analysis

#### Methodological testing

The COSMIN checklist for patient-reported outcome instruments provided a helpful framework for methodological testing [27]. Reliability, or whether a measurement is free from measurement error, was assessed through internal consistency and measurement error. Validity, the degree to which a questionnaire measures the construct it intends to measure, was assessed through measuring face validity, convergent/discriminant validity to the gold standard, and responsiveness to known groups.

Descriptive statistics were calculated to describe the sample, the QOL10 items’ characteristics, scores for the QOL10, QOL5, and QOL1, and the WHOQOL-BREF domain scores and single-item score. WHOQOL-BREF raw domain scores were transformed according to the WHO Group’s guidelines [3], yielding four domain scores on a 0–100 scale comparable to other World Health Organization QoL tools. Raw item scores from the QOL10, QOL5, and QOL1 were converted into a 0.1-0.9 scale where 0.1 corresponded to the worst rating and 0.9 to the best. Mean scores were calculated for health, ability, and self-assessed aspects of QoL, and composite scores were calculated as the mean of these three aspects for the QOL10 and QOL5 [19]. The question asking about one’s relationship to a partner was excluded in calculations for those who did not answer this question, as per the developers, as a lack of a “not applicable” option led to 63 missing answers for this item.

To determine whether the QOL10 measured multiple domains of QoL that could be validated against the domains of the WHOQOL-BREF, we used exploratory factor analysis to explore a possible factor structure. The data were tested to ensure they met the requirements for a factor analysis (FA) using the Kaiser-Meyer-Olkin measure of Sampling Adequacy and Bartlett’s test of sphericity [28]. An oblique rotation was chosen for the FA as we anticipated that the underlying factors would be related. A generalized least squares estimation procedure was used, as it is considered the most appropriate for our sample size of just over 100 [29, 30]. Goodness of fit was determined by *χ*^2^, wherein a non-significant *χ*^2^ suggests the factor model adequately describes the relationships among the variables [31]. The number of factors was determined using the FA and the number of factors to retain was determined using a scree plot and eigenvectors greater than 1. Ten item means and standard error were calculated for item analysis. Factor loadings of 0.4 or above were considered relevant [32], and any items with high loadings on multiple factors were assigned to their highest loaded factor. Missing data was excluded pairwise due to the large missing amount for the “partner” question.

Using the items determined relevant to each factor, subscales for the QOL10’s factors were derived using the same methodology as the WHOQOL-BREF’s domain scores. Missing data was also treated according to the WHO Group’s guidelines: a subscale was not calculated if any item was missing, as each factor contained less than six items (with the exception of the question asking about partners; the social subscale was calculated based on the four remaining questions for participants who did not answer the partner question). Cronbach’s α was calculated to evaluate the internal consistency of the subscales as well as the entire QOL5; α is not a useful test of multidimensional reliability and therefore not calculated for the entire QOL10.

Convergent/discriminant validity was tested through measuring Pearson’s product moment correlation (r) between composite QOL10, QOL5, and QOL1 scores, QOL10 subscales, and WHOQOL-BREF domains hypothesized to measure equivalent constructs, and through measuring the point-biserial correlation (r_pbc_) between known groups and the various QoL tools. We hypothesized that each score and subscale from the QOL10 would display convergent validity with the WHOQOL-BREF domains, and that the QOL10 scores would be able to discriminate between the known groups [15]. A p-value of 0.05 was used to determine statistical significance. Finally, a brief discussion of face validity explored the appropriate subjectivity of the QOL10’s items. SPSS version 22 was used for all statistics.

## Results

### QOL10 item statistics and composite score, and QOL5 and QOL1 scores

Responses to most items in the QOL10 skewed towards “good”, with this response being the most common for seven items (between 32.7 % and 55.6 %). Responses were higher for the “love” and “partner” items (most answered “very good”), and lower for the “overall QoL” item (most answered “neither good nor poor”).

Most items were missing very few answers – between 0 and 5 – with the exception of the “partner” question, which was not applicable for 63 participants. The mean composite score for the QOL10 was 0.59 (on a 0.1–0.9 scale), 0.60 for the QOL5, and 0.53 for the QOL1. Details are presented in Table 2.

#### WHOQOL-BREF domain scores and single-item score

The lowest-reported domain was physical health QoL (mean of 44.9, on a scale of 0–100), while the highest was social relationships QoL (62.3). Scores for psychological health QoL (54.6) and environment QoL (57.3) scores fell in-between. Most participants (36.4 %) responded “good” to the single item rating overall QoL, and the next largest proportion responded “neither good nor poor” (34.6 %), followed by “very good” (15.0 %), “poor” (13.1 %), and “very poor” (0.9 %). Cronbach’s alpha was acceptable for the physical health domain (.763), psychological health domain (.792), and environment domain (.762), but was somewhat lower for the social relationships domain (.541).

### Construct validity

#### Exploratory factor analysis

The Kaiser-Meyer-Olkin measure of Sampling Adequacy (KMO = 0.779) and Bartlett’s test of sphericity [*χ*^2^ (45) = 133.52, *P* < 0.001] indicated that the data were adequate for conducting a FA. The FA extracted two significant factors which together explained 56.0 % of the total variance. Eigenvalues were 3.57 and 1.20 before oblique rotation, and 2.85 and 2.72 after rotation. Factor loadings are presented in Table 3. The first factor of the two retained factors is interpreted as “social QoL”, and contains five items on one’s social functioning, ability to love, relationship to friends, sexual functioning, and relationship to partner. The second retained factor is interpreted as “global QoL” and includes five items on mental health, physical health, a global assessment of QoL (the latter question comprising the QOL1), working ability, and how one feels about oneself. The factors were moderately associated with one another (*r* = 0.339). Three out of ten items were associated with both factors, which is an overlap expected between two correlated factors. According to the *χ*^2^ goodness-of-fit test, the model provided a suitable fit.

**Table 3:** Factor loadings of the “QOL10” after exploratory factor analysis

	Factor 1: “social QoL”	Factor 2: “global QoL”
How do you consider your social functioning at the moment?	0.829	
How do you consider your ability to love at the moment?	0.664	
How are your relationships with your friends at the moment?	0.599	
How do you consider your sexual functioning at the moment?	0.577	
How is your relationship with your partner at the moment?	0.541	
How do you consider your mental health at the moment?	0.421	0.739
How would you assess your quality of your life now?	0.502	0.714
How do you consider your physical health at the moment?		0.711
How do you feel about yourself at the moment?	0.497	0.608
How is your working ability at the moment?		0.570
Goodness of fit: *χ*2 = 17.327, *d.f.* = 26, *p* = 0.899 *QoL: quality of life*		

Factor structure and loadings of the “QOL10” after exploratory factor analysis

Reliability was acceptable for both subscales of the QOL10 and for the QOL5 composite scale. Table 4 displays Cronbach’s α for each factor extracted: 0.814 for the “social QoL” factor and 0.771 for the “overall QoL” factor. Cronbach’s α for the QOL5 scale was 0.718.

**Table 4:** Descriptive statistics and reliability estimations of “QOL10” subscales calculated from the exploratory factor analysis

	N	Min	Max	Mean	s.e.m.	SD	Skewness	Kurtosis	Cronbach’s
Subscale 1: “social QoL”	100	10	100	57.9	2.06	20.6	-.02	-.56	0.814
Subscale 2: “global QoL”	102	5	100	58.5	1.90	19.3	-.41	-.27	0.771

Descriptive statistics and reliability estimations of “QOL10’s” social and global subscales calculated from the exploratory factor analysis

*QoL* quality of life

#### Association of QoL scores with WHOQOL-BREF scores and known groups

Table 5 shows how each QOL10 composite score and subscale correlates with the gold measure standard and known groups. As hypothesized, the QOL10’s single-item measure of global QoL, the QOL1, correlated strongly with the WHOQOL-BREF’s single-item measure (*r* = 0.671, *p* < 0.001), as did the QOL5 and QOL10. The QOL10, QOL5, and QOL1 were also strongly correlated to each domain of the WHOQOL-BREF (between *r* = 0.382 and *r* = 0.768, *p* < 0.001). Our hypotheses about subscale correlations to the gold standard were also met. The highest correlation between the “social QoL” subscale was with the social relationships domain (*r* = 0.680, *p* < 0.001), suggesting appropriate convergent validity with this domain.

**Table 5:** Correlations between quality of life scales, WHOQOL-BREF domains, and known groups

	QOL10			QOL5	QOL1
	QOL10	Exploratory factor analysis			
		Subscale 1: “social QoL”	Subscale 2: “global QoL”		
*WHOQOL-BREF scores* ^*a*^					
Physical health QoL	.622***	.344***	.707***	.596***	.495***
Psychological health QoL	.768***	.490***	.734***	.772***	.676***
Social QoL	.629***	.680***	.499***	.548***	.495***
Environment QOL	.436***	.244*	.452***	.382***	.501***
Overall QoL item	.623***	.448***	.576***	.486***	.671***
*Known groups* ^*b*^					
Additional chronic disease	.002	.116	-.006	-.015	-.041
Clinical anxiety symptoms	-.532**	-.251*	-.509***	-.534***	-.410***
Clinical depression symptoms	-.497**	-.173	-.484***	-.510***	-.425***
Physical inactivity	-.369**	-.094	-.392***	-.373***	-.411***
Psychiatric services utilization, past year	-.119	.047	-.137	-.114	-.012

*p < 0.05; **p < 0.01, ***p < .001

^a^Numbers are Pearson’s correlations (r); ^b^numbers are point-biserial correlations (r_pbc_)

*QoL* quality of life

Convergent validity testing: Correlations between “QOL10” composite score and subscales, “QOL5”, “QOL1”, WHOQOL-BREF domains, and known groups

Our hypotheses regarding the QOL10’s ability to discriminate between groups previously reported to have differential global QoL, were partly confirmed. QOL10, QOL5, and QOL1 scores were negatively related with exhibiting symptoms of clinical anxiety, clinical depression, and being physically inactive, but did not distinguish between the remaining two known groups, namely, psychiatric services utilization and additional chronic somatic disease. The global subscale’s correlations with known groups followed the same pattern as among the QOL10, QOL5, and QOL1. The social subscale correlated only with those exhibiting symptoms of clinical anxiety (*r* = −0.251, *p* < 0.05).

#### Face validity

Each item in the QOL10 elicits self-evaluations of various aspects of QoL (e.g., item 4, “how are your relationships with your friends?”) rather than attempting to measure their quality objectively (e.g., “to what extent can you confide in your friends?”) or quantifying them (“how many friends do you have?”). The QOL10’s wording allows the respondent to consider, in this example, whichever functions s/he expects to be performed by friends, to evaluate these relationships based on global friendship expectations or on individual expectations, to compare them to past performance, and so on [33].

## Discussion

In this paper, we validated the “QOL10” for the first time against the gold standard of the WHOQOL-BREF, in a sample of 107 participants in a substance use disorder treatment study. We also confirmed the validity of the embedded “QOL5” questionnaire and single-item “QOL1” in measuring global quality of life (QoL). The QOL10 provides short, clinically useful measures of social QoL and global QoL via two subscales. The social subscale is of particular utility, as social QoL is rarely explored among this population but appears to be conceptually distinct from global QoL. Our findings also suggest that social QoL is less influenced by typically-measured health, substance and treatment-specific factors. Social QoL may “respond” differently than global QoL to typical treatment practices, pointing towards the importance of interventions targeted specifically at the social lives of the SUD population. The first step in designing such interventions is introducing patients’ social lives as priorities in the clinical encounter, which can be accomplished through the administration of a short QoL measure such as the QOL10.

WHOQOL-BREF domain score patterns were similar to previous findings internationally and in Norway [34, 35]. Based on strong correlations with the WHOQOL-BREF and sensitivity to three out of five known groups [25, 36] the QOL10, QOL5, and QOL1 tools appear to provide valid measurements of global QoL. The lack of clear relationships to patients in the remaining two groups is not surprising [5], and speaks to the complexity of QoL, particularly among this population with a constellation of vulnerabilities.

Despite the variety of vulnerabilities, and strengths, with which patients present to treatment, focus is often predominantly given to factors deemed most relevant to the development or amelioration of SUD [14]. A main clinical strength of the QOL10 is therefore its social QoL subscale and the attention it draws first to patients’ social lives, and second to patients’ own evaluations. Utilizing subjective evaluations to measure the social wellbeing of persons with a SUD reveals more complex relationships between common clinical concerns and how dis/satisfied persons are with their social lives – perhaps an explanation as to why the social QoL subscale showed no relationship to psychiatric service utilization, additional chronic somatic disease, depression, or physical activity.

Patient evaluations of the quality of their social lives, such as those elicited by the QOL10, are likely more important to understanding wellbeing than objective measures of social life, whether quantitative or qualitative [37]. The social subscale of the QOL10 provides respondents with a unique opportunity to evaluate their relationships with their friends, family, and partners, and to reflect on their social and sex lives, without the tool including predetermined expectations of a certain amount of these actors or particular roles they ought to play. This subjectivity is a prerequisite to a good QoL measure and the social subscale is as such a useful tool to tap into this dimension of SUD patients’ lives.

In this validation study, the common clinical concerns represented by the known groups included may not predict the health of respondents’ social lives, which we know influence treatment trajectories and outcomes [38]. This reminds us that it is both possible and worthwhile in treatment to spend attention on strengthening the social lives of patients who might otherwise be assumed to have other, more urgent needs or priorities. Alternatively, the QOL10 might have lacked sensitivity to two of the known groups because the burdens of those conditions were relatively homogenous across the sample. The validation-nature of this study, however, limits further discussion of known group correlation, as the study was not intended to provide a sophisticated analysis of factors influencing QoL. Further exploration of the QOL10 should include an explicit subgroup analysis between men and women, as women report receiving less social support than men, social support being provided more by substance users than non-users, and providing more support to other substance users than men provide [39–41]. Future research should also explore clinical and under-reported correlates of social QoL, particularly to find out how to support patients’ social health. The QOL10 may be improved by adding “not applicable” responses, particularly to the partner question, so that inapplicable questions are not considered missing values. High quality test-retest reliability assessment as well as further psychometric testing should also be conducted [42].

## Conclusion

This validation study found the “QOL10”, “QOL5”, and “QOL1” to have sufficiently high correlations to the WHOQOL-BREF and selected known groups to conclude that each tool measures global QoL. The QOL10 discriminates as well as the previously validated QOL5 and QOL1. The QOL10 should be utilized in a substance use disorder treatment context for its two subscales capturing global QoL and the more novel social QoL. Given the social subscale’s high internal validity, we additionally suggest that it can be a psychometrically stronger alternative to the WHOQOL-BREF’s social relationships domain [43]. The QOL10 measures global and social QoL constructs on par with WHO recommendations, and practitioners interested in these constructs may find the QOL10 a shorter and more robust tool than the WHOQOL-BREF. Our findings support the inclusion of a distinct social domain into QoL research [1] among this population, beyond simply social relationships, as the WHOQOL-BREF measures. The social subscale of the QOL10 provides patients with the rare opportunity to evaluate their social lives and provides a reflection of these evaluations for a clinical context, rather than reflecting researcher-determined priorities. The single-item QOL1 and overall QOL5 tools were also deemed valid measurements of global QoL.

## Abbreviations

FA: factor analysis
NorComt: Norwegian Cohort of Patients in Opioid Maintenance Treatment
OMT: opioid maintenance treatment
QoL: quality of life
SUD: substance use disorder
